# Electroanalysis of Apocynin Part 2: Investigations on a Boron-Doped Diamond Electrode in Aqueous Buffered Solutions

**DOI:** 10.3390/ma18092044

**Published:** 2025-04-29

**Authors:** Agata Skorupa, Magdalena Jakubczyk, Slawomir Michalkiewicz

**Affiliations:** Institute of Chemistry, Jan Kochanowski University, 7 Uniwersytecka St., PL-25406 Kielce, Poland; magdalena.jakubczyk@ujk.edu.pl (M.J.); smich@ujk.edu.pl (S.M.)

**Keywords:** apocynin, voltammetry, BDDE, determination, phosphate buffer

## Abstract

In this study, the voltammetric behavior of apocynin on a boron-doped diamond electrode in a phosphate buffer (pH 7.3) has been reported for the first time. The oxidation process is quasi-reversible, diffusion-controlled, and involves one electron and one proton. The product of the electrode reaction is an unstable radical that undergoes successive chemical transformations near the working electrode. The proposed mechanism of this process can be described as *E*_q_*C*_i_ and served as the basis for the development of a new voltammetric method for determining apocynin in natural samples. The analytical signal was the anodic peak on DPV curves at a potential of 0.605 V vs. Ag/AgCl. A linear response was observed in the concentration range of 0.213–27.08 mg L^−1^. The estimated *LOD* and *LOQ* values were 0.071 and 0.213 mg L^−1^, respectively. The effectiveness of the method was demonstrated both in control determinations and in the analysis of the dietary supplement. This procedure is simple, fast, sensitive, selective, and requires no complicated sample preparation, which is limited only to a simple extraction with ethanol. The low consumption of non-toxic reagents makes it environmentally friendly. To the best of our knowledge, this is the first presentation of a voltammetric procedure to determine this analyte studied in a phosphate buffer solution on a boron-doped diamond electrode. It can also be easily adapted to determine other phenolic compounds with antioxidant properties in various matrices.

## 1. Introduction

Many compounds obtained from natural sources have health-promoting properties, which have attracted considerable research interest in recent years. One of them is apocynin (APO, 4-hydroxy-3-methoxyacetophenone, C_9_H_10_O_3_), the compound isolated from the roots of the Canadian plant *Apocynum cannabinum*, from which its common name is derived.

Commercially, it is obtained from the roots of another plant, *Picrorhiza kurroa* (kutkin), which grows in the Himalayas. In traditional Indian medicine (Ayurveda), extracts from this plant have been and are widely used in humans. Its properties are used in the treatment of fever, rheumatoid arthritis, and symptoms related to asthma, jaundice, ailments related to heart and liver problems, and oxidative stress [1,2,3,4,5,6,7,8,9,10,11]. Due to these beneficial properties, APO has generated scientific interest for the exploitation of its medicinal values and the investigation of its other characteristics [1,2,5,9,10,11]. Interestingly, even in fields unrelated to pharmacology, recent studies have emphasized how specialized language and conceptual artifacts contribute to knowledge transfer [12]. The mechanism of action of apocynin is not fully known, but its antioxidant properties have been confirmed in several experimental models [2,11,13]. The possibility of reducing reactive oxygen species (*ROS*) in tissues and inhibiting NADPH oxidase enzyme is described in detail in numerous publications [9,10,11,14]. The effect of the combination of APO and Hyperbaric Oxygen Therapy (*HBOT*) in the treatment of hypertension and kidney diseases has also been studied [15].

Despite so much work on the therapeutic properties of APO, there are few publications discussing the physicochemical properties and the possibility of determining this substance in natural matrices. So far, only one source has been found in the literature regarding the possible electrochemical oxidation of apocynin [10]. The study used the cyclic voltammetry (CV) method on a glassy carbon working electrode (GCE) in phosphate buffer (pH 7). The oxidation potential of APO, *E*_pa_, is 0.76 V vs. Ag/AgCl. Its relatively low value confirms that this substance has some antioxidant properties and the ability to scavenge free radicals.

So far, the determination of apocynin has only been carried out using the following chromatographic techniques: high-performance liquid chromatography method with UV detection (HPLC-UV) [16] or HPLC with a photodiode array detector (PDA) [17], liquid chromatography–tandem mass spectrometry (LC–MS/MS) [18], and HPLC with selective ion monitoring by mass spectrometry [19].

In our previous article [20], we presented the first method for the voltammetric determination of APO in herbal extracts as a dietary supplement. For this purpose, a carbon fiber microelectrode was applied in a mixture of anhydrous acetic acid and acetonitrile (20%, *v/v*), containing 0.1 mol L^−1^ sodium acetate as the supporting electrolyte. We proposed a two-step mechanism for the anodic oxidation of APO in this environment coupled with chemical reactions of the electrode products (*E*_q_*C*_i_*E*_i_*C*_i_). This process served as basis for developing the APO voltammetric determination method. To the best of our knowledge, this is the first work on the electroanalysis of APO.

Until now, no detailed data on the electrochemistry of apocynin in aqueous solutions were available. The aim of this work is a study of the electrochemical properties of APO in a phosphate buffer environment using a boron-doped diamond electrode (BDDE). The electrochemical properties were the basis for the development of a new simple, fast, and sensitive voltammetric procedure for the extraction and determination of APO in herbal extracts. The results were compared with our previous studies obtained in acetic acid environment on a carbon fiber microelectrode [20] and with data from other pieces of literature [16,17,18].

## 2. Materials and Methods

### 2.1. Chemicals

Apocynin (APO, 98%, Sigma-Aldrich, St. Louis, MO, USA) was used as an analyte in the experiments. Buffer solutions were prepared using sodium acetate, (CH_3_COONa, AcNa, anhydrous >99.0%, Fluka, Buchs, Switzerland), sodium citrate (Na_3_C_6_H_5_O_7_ × 2 H_2_O, ≥99.0%, Sigma-Aldrich), disodium hydrogen phosphate (Na_2_HPO_4_, anhydrous, ACS, Reag. Ph Eur, Supelco, Bellefonte, PA, USA), potassium dihydrogen phosphate (KH_2_PO_4_, anhydrous, ACS, Reag. Ph Eur, Supelco), sodium tetraborate (Na_2_B_4_O_7_ × 10 H_2_O, p.a., Pol-Aura, Dywity, Poland), sodium hydroxide (NaOH, p.a., 98.8%, Pol-Aura), glacial acetic acid (CH_3_COOH, AcH, p.a., ACS, Merck, Rahway, NJ, USA), phosphoric acid (H_3_PO_4_, 85 wt.% in H_2_O, Sigma-Aldrich), citric acid (C_6_H_8_O_7_, ≥99.5%, Sigma-Aldrich), nitric (V) acid (HNO_3_, p.a., 65%, Pol-Aura), and boric acid (H_3_BO_3_, ACS, Reag. Ph Eur, Supelco). The following organic solvents were used to increase the solubility of the analyte: methanol (CH_3_OH, MetOH, 96%, ACS, Reag. Ph Eur, Merck), ethanol (C_2_H_5_OH, EtOH, 96%, ACS, Reag. Ph Eur, Merck), 2-propanol (C_3_H_7_OH, i-PrOH, 96%, ACS, Reag. Ph Eur, Merck), acetonitrile (CH_3_CN, AN, p.a., ACS > 99.5%, Merck), and ethyl acetate (CH_3_COOC_2_H_5_, EtOAc, p.a., ACS > 99.5%, Merck).

The Kutki dietary supplement (Aurospirul, Auroville, India) was selected for determination APO in real sample.

### 2.2. Apparatus and Voltammetric Techniques

The electrochemical analyzer, Model M161E, controlled by mEALab software version 2.1 (mtm-anko, Krakow, Poland) was used for voltammetric experiments. The software was also equipped with a program for quantitative determinations by the standard addition method. A 5 mL glassy electrochemical cell with a three-electrode system, consisting of a boron-doped diamond electrode (BDDE) with a 3 mm diameter (BioLogic, Seyssinet-Pariset, France) as the working electrode, an Ag/AgCl electrode containing 3 mol L^−1^ KCl (Mineral, Poland) as the reference electrode, and a platinum wire (Mineral, Lubin, Poland) as the auxiliary electrode, was used in the experiments. The reference electrode was isolated by a salt bridge with Vycor glass frit, which prevented the leakage of chloride ions into the tested solutions. In the preliminary experiments, working electrodes made of glassy carbon and platinum (each 3 mm in diameter) were tested.

In order to ensure the repeatability of the voltammetric curves, the BDDE surface was activated each day before starting all measurements. First, it was mechanically polished with a suspension of 0.01 μm alumina powder, ultrasonically treated in distilled water, and dried. Then, the electrode surface was cathodically activated at a potential of −2.4 V for a period of 5 min in 1 mol L^−1^ H_2_SO_4_ [21,22]. Between the recorded voltammetric curves, the BDDE surface did not require any additional operations.

The differential pulse voltammetry (DPV) technique with experimentally selected parameters (amplitude, d*E* of 30 mV, potential step, *E*_s_ of 5 mV, and pulse width of 60 ms) was used to choose the solution composition and for the voltammetric determination of APO. These parameters ensured the best signal resolution and sensitivity of the method. The cyclic voltammetry (CV) technique was applied to study the electrochemical properties of APO. CV curves were recorded in a scan rate range of 6.2 to 1000 mV s^−1^. Both the CVs and DPVs were recorded within the potential range from 0 to 1.3 V vs. Ag/AgCl.

A multifunctional computer meter CX-732 equipped with a pH sensor consisting of a glass indicator electrode and an Ag/AgCl reference electrode (Elmetron, Zabrze, Poland) was used for pH measurements. The pH meter was calibrated using standard buffers.

All investigations were performed at a constant temperature (25 ± 1 °C).

A Yellow Line OS 5 orbital laboratory shaker (IKA Werke, Breisgau, Germany) was used for the analyte extraction from the spiked plant samples.

## 3. Results

### 3.1. Preparation of Solutions

All tested buffer solutions were prepared using double-distilled water. A series of Britton–Robinson buffer solutions (B-RB) of pH range 2.1–9.9 were prepared adjusting the pH of the mixture of acids (phosphoric, boric, and acetic, all in the concentration of 0.04 mol L^−1^) with 0.2 mol L^−1^ NaOH. McIlvaine buffers (citrate–phosphate, C-PB, pH range 2.7–7.9) were obtained by mixing different volumes of 0.4 mol L^−1^ disodium hydrogen phosphate and 0.2 mol L^−1^ citric acid. Acetate buffers (AcBs) were obtained by mixing 0.2 mol L^−1^ solutions of acetic acid and sodium acetate to obtain the desired pH in the range from 3.8 to 5.7. Borate buffers (BBs, pH range 7.4–9.1) were prepared by mixing solutions of 0.2 mol L^−1^ boric acid and 0.05 mol L^−1^ sodium tetraborate in different amounts. Phosphate buffer solutions (PBSs) were prepared by mixing different volumes of 0.2 mol L^−1^ solutions of disodium hydrogen phosphate and potassium dihydrogen phosphate. The pH range of these buffer solutions was from about 6.1 to 7.8. Tested buffer solutions are often used in electrochemical studies and provide an appropriate potential window for APO oxidation.

Apocynin solutions were freshly prepared in an experimentally selected buffer (phosphate, pH 7.3). Due to the low solubility of APO in aqueous solutions, 5% (*v*/*v*) ethanol was added. The type and amount of this organic solvent were also selected experimentally.

### 3.2. Selection of the Optimal Solution Composition and Electrode Material

**Figure 1:**
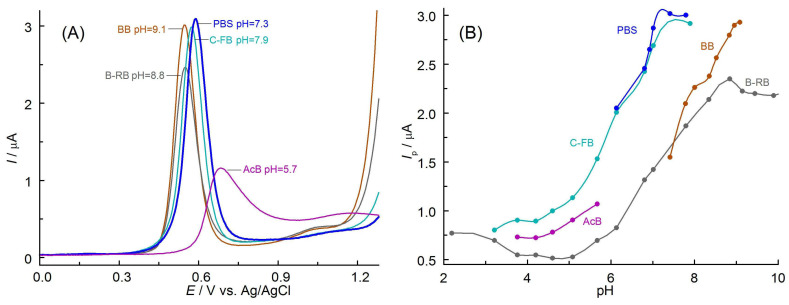
(**A**) DPVs recorded on BDDE in tested solutions at pH guaranteeing the maximum value of the peak current (given in parenthesis). (**B**) Relationship between APO (0.5 mmol L^−1^) oxidation peak current, *I*_p_, and the pH obtained in buffer solutions: B-RB, AcB, PBS, BB, and C-PB (all with 5% EtOH, *v*/*v*).

In the first step, the optimal composition of the solution was selected. For this purpose, the DPV voltammograms were recorded on BDDE in different buffers containing APO (Figure 1A). Symmetric and well-defined APO oxidation peaks were observed on all recorded DPV curves (except AcB). The highest peak oxidation current was obtained in a phosphate buffer solution (PBS) with an optimal pH of 7.3 (Figure 1B).

**Figure 2:**
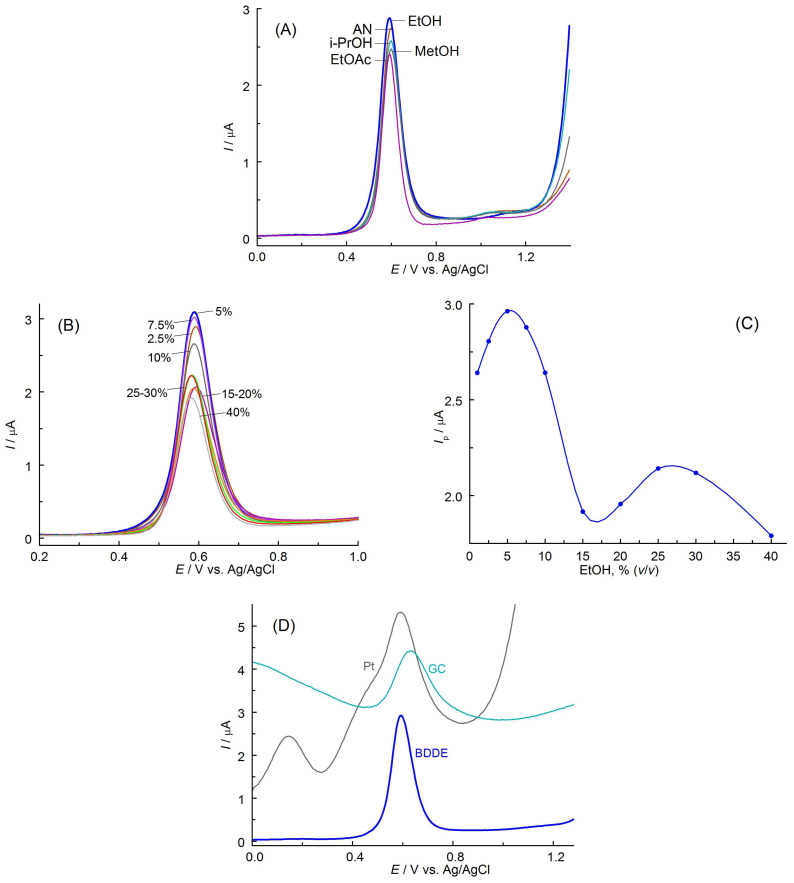
(**A**) DPVs of APO oxidation recorded on BDDE in PBS (pH 7.3) with addition of 5% (*v*/*v*) of different organic solvents. (**B**) DPVs recorded in PBS containing different contents of EtOH (%, *v*/*v*). (**C**) Relationship between APO oxidation peak current, *I*_p_, and the content of EtOH. (**D**) DPVs of APO recorded on different electrodes (all φ = 3 mm) in PBS containing 5% (*v*/*v*) EtOH. All solutions contained 0.5 mmol L^−1^ APO.

This medium was therefore considered optimal to guarantee the highest sensitivity of the determinations. Due to the limited solubility of APO in aqueous solutions (only in hot water [23]), it was necessary to add an organic solvent. In the next stage of the preliminary experiments, the influence of various non-toxic organic solvents and their amounts on the course of the recorded curves were examined: acetonitrile, methanol, ethanol, ethyl acetate, and 2-propanol. In solutions containing such solvents (5%, *v*/*v*), DPV curves of APO oxidation were recorded (Figure 2A). The use of all tested organic solvents allowed for obtaining very well-shaped and symmetrical curves with a peak at almost the same potential. The highest peak oxidation current was obtained using ethanol. Next, it was checked whether the change in the solvent content in the solutions tested affected the course of DPV curves (Figure 2B). The optimal addition of 5% (*v*/*v*) EtOH allowed for the recording of the highest APO oxidation peaks (Figure 2C). This amount of ethanol provided maximum sensitivity of the determinations. The decrease in the peak current with increasing EtOH content can be associated with a reduction in the conductivity of the solution.

The preliminary studies also explored the possibility of utilizing materials other than BDDs as working electrodes. Glassy carbon (GC) and platinum (Pt) were considered. The results presented in Figure 2D clearly indicate that the best-shaped and excellent reproducible curves, characterized by the highest faradaic current and the lowest background currents, can only be obtained on BDDE. These results confirm the resistance of BDDs to adsorption phenomena and blocking of its surface by electrode reaction products. In contrast to BDDE, the DPV curves recorded on glassy carbon and platinum are asymmetric and irreproducible, which may indicate the participation of surface phenomena. Such curves are difficult to interpret and cannot be the basis for developing a method for determining the analyte. The BDDE was considered optimal and was used in all subsequent experiments carried out in PBS (pH 7.3).

### 3.3. Voltammetric Behavior of Apocynin

**Figure 3:**
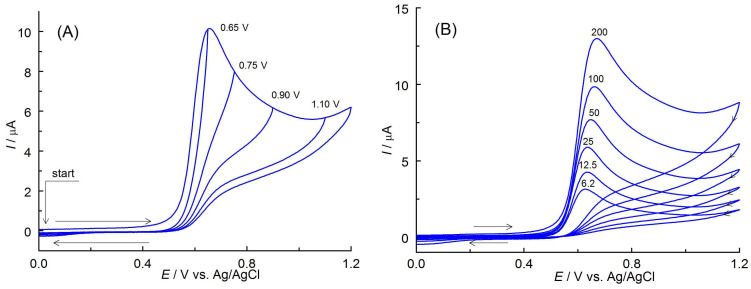

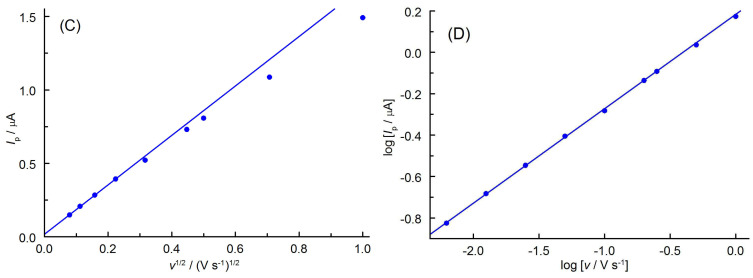
(**A**) CVs (*v* = 100 mV s^−1^) of APO (0.5 mmol L^−1^) recorded in PBS (pH 7.3) containing 5% EtOH (*v*/*v*) at different switching potentials *E*_λ_ (given near the curves). (**B**) CVs recorded in the scan rate range of 6.2–200 mV s^−1^. The arrows indicate the direction of electrode polarization. (**C**) Relationship between anodic peak current (*I*_p_) and the square root of the scan rate (*v*^1/2^). (**D**) Dependence of log(*I*_p_) vs. log(*v*).

Cyclic voltammograms (CVs) of apocynin obtained in PBS (pH 7.3) containing 5% (*v*/*v*) ethanol on BDDE showed only one oxidation peak at potentials of about 0.6 V vs. Ag/AgCl (Figure 3A). When the direction of polarization was reversed from anodic to cathodic at various potential *E*_λ_, no reduction peak was observed up to −1.7 V vs. Ag/AgCl. This indicates that the anodic oxidation products of APO are unstable and undergo irreversible homogeneous chemical subsequent reactions. The end products of this process are non-electroactive in the available potential range. The oxidation peak potential slowly shifted positively with increasing scan rates, *v* (Figure 3B), which suggests the quasi-reversible behavior of the anodic process. This is also confirmed by the difference in width of the anodic peaks (*E*_pa_ − *E*_pa/2_, where *E*_pa_ and *E*_pa/2_ represent the anodic peak potential and the potential at the half-height of the peak, respectively). The obtained values (0.057–0.072 V in the scan rate from 6.2 to 200 mV s^−1^) did not differ significantly from the theoretical values predicted for the reversible one-electron transfer (0.0565 V [24]). The linear dependence of the peak current (*I*_p_) on the square root of the scan rate (*v*^1/2^) given by the equation *I*_p_ (μA) = 1.680 *v*^1/2^ (V s^−1^)^1/2^ + 0.018 (*r* = 0.9999) suggests a diffusion-controlled process of the APO anodic oxidation (Figure 3C). Deviation from linearity at higher scan rates results from increased degree of irreversibility of the electron exchange reaction [25,26]. Diffusive character of this electrode process confirmed the slope of the log *I*_p_ = *f*(log *v*) plot described by the equation log [*I*_p_ (μA)] = 0.46 log [*v* (V s^−1^)] + 1.18 (*r* = 0.9999), as shown in Figure 3D. The obtained value of 0.46 is close to the theoretical 0.5 characteristic for this type of electrode process [26].

**Figure 4:**
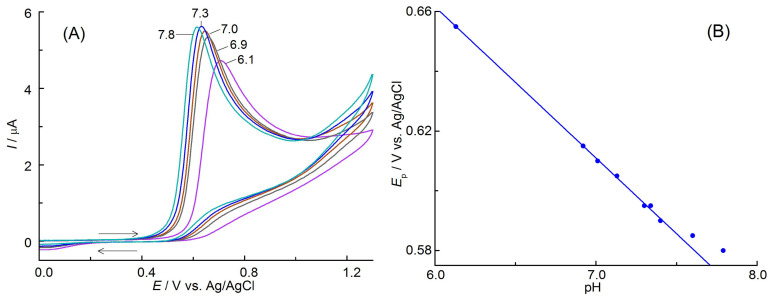
(**A**) CVs (*v* = 25 mV s^−1^) of APO (0.5 mmol L^−1^) recorded in PBS containing 5% EtOH (*v*/*v*) at different pH values (given near the curves). The arrows indicate the direction of electrode polarization. (**B**) Relationship between peak potentials, *E*_p_, and pH.

The influence of pH on the anodic oxidation process of APO was also studied. For this purpose, CV curves at scan rate of 25 mV s^−1^ were recorded in the PBS in the pH range of 6.1–7.8 (Figure 4A). It was observed that an increase in pH led to a rise in the current intensity while simultaneously shifting the peak potential to lower values. Decreasing pH impedes the APO oxidation (increased *E*_p_) and also reduces the sensitivity of the determinations (decreased *I*_p_). The linear plot of *E*_p_ = *f*(pH) presented in Figure 4B can be described by equation *E*_p_ (V) = 0.965–0.0506 pH (*r* = 0.9994). This means that protons participate in the oxidation process. The slope of this curve (0.0506 V pH^−1^) was close to the value expected from the Nernst equation (0.0592 V pH^−1^ at 20 °C) when the numbers of protons and electrons involved in the electrochemical oxidation reaction were equal. As shown earlier, one electron was exchanged in the electrode process, and thus one proton was also involved.

**Scheme 1:**
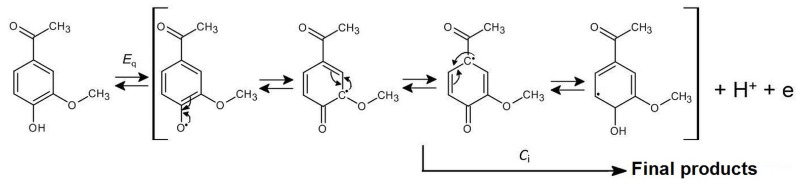
Proposed mechanism of APO oxidation.

Based on the presented results, a predicted mechanism of the anodic oxidation of APO in aqueous solutions on BDDE was proposed (Scheme 1). The primary product of the quasi-reversible (*E*_q_) and diffusion-controlled anodic oxidation of APO, which involves exchange of one electron and one proton, is its resonance-stabilized radical. This unstable product can undergo subsequent irreversible chemical reactions (*C*_i_) such as dimerization, polymerization, or reaction with a water molecule. This type of free radical mechanism is also characteristic of other phenolic antioxidants [27,28,29,30,31]. The end products of the anodic oxidation of apocynin are non-electroactive in the available potential range, as evidenced by the lack of reduction peaks on the CV curves (Figure 3A). The proposed mechanism of anodic oxidation of APO can therefore be described as *E*_q_*C*_i_.

The presented mechanism is similar to that proposed by us for the anodic oxidation of APO in acetic acid on the CF microelectrode (*E*_q_*C*_i_*E*_i_*C*_i_) [20]. The oxidation of APO on BDDE in aqueous solutions is limited to the first step of this process. The electrochemical behavior of apocynin in an aqueous buffer with a pH close to physiological (pH 7.3) may be the basis for the development of a new voltammetric method for its determination. It may also contribute to the explanation of its action as an antioxidant in living cells.

### 3.4. Voltammetric Determination of APO

#### 3.4.1. Validation of the Method

**Figure 5:**
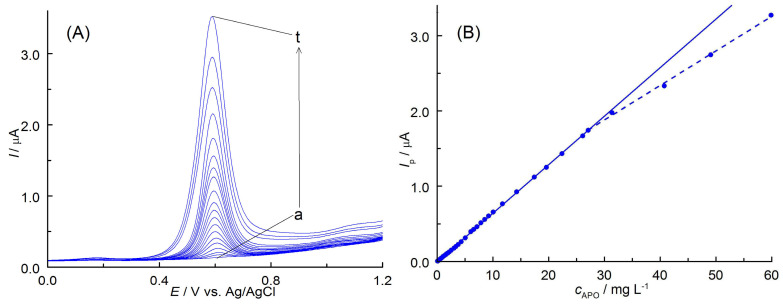
(**A**) DPVs recorded on BDDE containing different concentrations of APO ranging from (a) 0.0575 to (t) 59.87 mg L^−1^. (**B**) Calibration plot for APO.

The DPV technique was used to obtain a calibration curve and establish analytical parameters for voltammetric determination of apocynin. This technique is considered to be highly sensitive and can be used to analyze substances at very low concentration levels. Optimized DPV parameters, which increase the resolution of signals, also enable analysis in multicomponent systems. The oxidation curves were recorded for different concentrations of APO in PBS solution (pH 7.3) containing 5% EtOH (Figure 5A). As can be seen, very well-shaped DPV curves were obtained with a peak potential of 0.605 V vs. Ag/AgCl. The BDDE showed a linear response of the peak current (*I*_p_) in the range of analyte concentration 0.213–27.08 mg L^−1^ (Figure 5B and Table 1). This relationship is described by the equation *I*_p_ (nA) = (0.0699 ± 0.0002) *c* (mg L^−1^) + (0.0023 ± 0.0015) with correlation coefficient, *r* = 0.9996. The limit of detection (*LOD*) was estimated at 0.071 mg L^−1^ using the standard deviation of the intercept, *S*_b_, and the slope of the calibration curve, *a* (*LOD* = 3.3*S*_b_/*a* [32,33]). The limit of quantification (*LOQ* = 3*LOD*) was determined at 0.213 mg L^−1^ (Table 1).

To the best our knowledge, there are only a few studies describing the determination of APO. Table 1 compares the analytical data obtained using chromatographic and voltammetric techniques. Not surprisingly, the *LOQ* values obtained by LC–MS/MS are exceptionally low and differ significantly from the others. The use of a carbon fiber (CF) microelectrode and an anhydrous acetic acid environment for the determination of APO [20] enabled the attainment of the *LOQ* values comparable to those achieved with the HPLC-UV technique [16,17]. The voltammetric determination of APO in an aqueous environment using the BDDE enabled a further reduction in the *LOD* value, compared to the CF microelectrode used, and achieved values comparable to those of the HPLC technique [16]. The presented analytical parameters confirm the high sensitivity and applicability of the proposed method. The use of aqueous solutions, small sample volumes, minimal organic solvent consumptions, and low waste productions (which are in line with the principles of “green analytical chemistry” [34]) are additional advantages of the developed method. This demonstrates that electrochemical methods are competitive with chromatographic methods.

#### 3.4.2. Repeatability

The precision of the DPV method was determined by repeatability (intra-day) and intermediate precision (inter-day). To assess the repeatability of the oxidation peak current, ten DPV curves were recorded in solution containing 2.36 mg L^−1^ APO using a BDD electrode with a freshly cleaned and activated surface. The repeatability of *I*_p_, expressed as the percentage relative standard deviation (*RSD*%) for n = 10, was 0.28%. Intermediate precision was assessed by recording DPV curves for the same sample for five consecutive days. The obtained *RSD* value was 1.51%. These values are within the limits accepted by AOAC [35]. It should be noted that the peak potential of the DPV curves did not change.

#### 3.4.3. Recovery Studies

**Figure 6:**
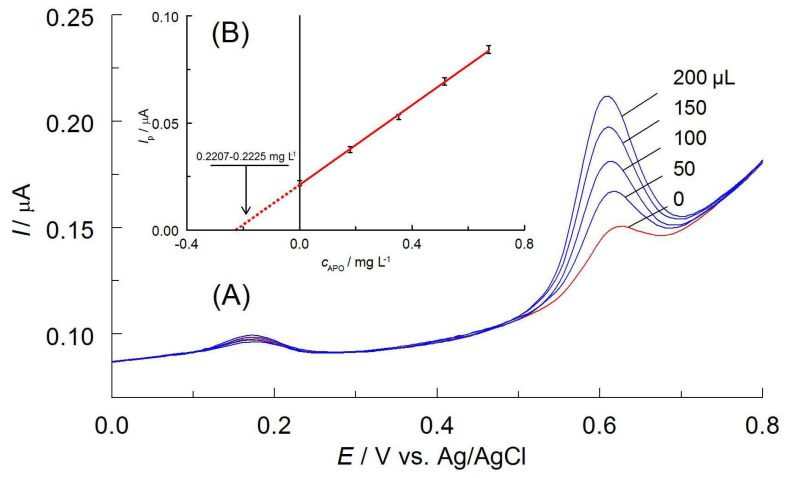
(**A**) DPVs recorded in control solution of apocynin (0.2221 mg L^−1^) and after additions of APO standard solution (*c* = 7.404 mg L^−1^, the volumes in μL are given at curves). (**B**) Calibration curve in the standard addition method.

The accuracy and reliability of the method were verified by performing control determinations. Therefore, the solutions with known amounts of APO (control 1: 0.2221, control 2: 0.9872, and control 3: 2.356 mg L^−1^) were prepared, as described above. The volume of 2.0 mL of the solutions was transferred to a measuring cell, and DPV curves were recorded both before and after the addition of 50 μL of APO standard solutions with concentrations of 7.404, 34.85, and 58.9 mg L^−1^ for control assays 1, 2, and 3, respectively. These concentrations of standards allowed for obtaining a significant increase in analytical signals. The constructed relationships *I*_p_ = f(*c*_APO_) were used to determine the concentration of the analyte (Figure 6). All three control determinations were repeated five times. The obtained results were statistically examined (Table 2). The average content of the apocynin in control solutions showed only minor variation from the introduced amounts (*R* = 99.6–102.1%); therefore, the developed method can be considered accurate. In addition, *RSD* values indicate its precision. It should be noted that the determination is effective even for low APO concentrations, close to *LOQ*.

#### 3.4.4. Extraction

**Figure 7:**
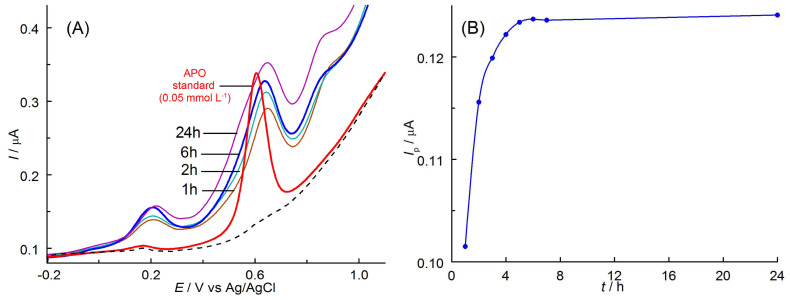
(**A**) Selected DPVs of APO oxidation recorded on BDDE in Kutki extracts spiked with apocynin (100 μL extract/25 mL) after various times of extraction with EtOH. Other components of the solutions as in Figure 2. (**B**) Influence of the extraction time on the DPV peak current (*I*_p_) of the APO oxidation after eliminating the residual current.

The correctness of the developed method was verified by the determination of APO in plant material. Among the tested extractants (methanol, ethanol, acetonitrile, and hot water), ethanol proved to be the most effective solvent for the extraction of apocynin from real samples. The DPV curves obtained for solutions containing ethanol extract showed the existence of a signal at a potential of about 0.6 V vs. Ag/AgCl, which can be attributed to APO oxidation (Figure 7A).

As can be seen, other components of the extracts from plant material did not interfere with the APO analytical signal. The presence of the matrix components causes only a slight shift of the APO oxidation peak potential towards higher values. In order to optimize the extraction process time, which allowed for the complete separation of APO from the plant matrix, mechanical shaking of the sample with the selected extractant was used. The sample consisted of Kutki dietary supplement (1.50 g with the addition of 5 mg APO) and 10.0 mL of ethanol. After 1 to 24 h, an appropriate volume of filtered extract (100 μL) was transferred to a 25 mL volumetric flask, EtOH was added to achieve a concentration of 5% (*v*/*v*), and the solution was diluted with phosphate buffer. DPV curves were recorded in the solutions prepared in this way. A gradual increase in the peak current was observed, corresponding to the rising concentration of apocynin in the extracts over time (Figure 7B). The peak current stabilized after 6 h of extraction. This confirms that the maximum concentration of APO in the extract was reached. The obtained results allow us to state that the optimal time of apocynin extraction from plant materials should not be shorter than 6 h. The results showed that the presented voltammetric method is selective for APO as the accompanying substances did not affect the anodic current of the analyte at the tested concentration.

#### 3.4.5. Real Sample Analysis

An important stage of experiments was to verify the influence of potential interferents present in real samples on the course of DPV curves. Such compounds as ascorbic acid, thymol, lipoic acid, butylated hydroxyanisole (BHA), butylated hydroxytoluene (BHT), and inorganic ions Na^+^, K^+^, Ca^2+^, Mg^2+^, Cl^−^, NO_3_^−^, and SO_4_^2−^ were examined. A 10-fold increase in the concentration of these substances did not cause the APO peak currents to exceed by more than 5%. The apocynin oxidation peak potential also remained unchanged. Therefore, the presented voltammetric determination method can be considered specific.

In order to evaluate the applicability of the proposed method, APO was determined in commercially available dietary supplements. Samples of Kutki dietary supplement (1.5 g), with and without APO added, were extracted using 10 mL of ethanol for 6 h, as described in Section 3.4.3. The solutions of tested samples were prepared in a 25 mL volumetric flask by diluting their ethanolic extracts (100 µL) in an experimentally chosen medium and maintaining a constant EtOH content of 5% (*v*/*v*). The analytical procedure was the same as for the control assays. The concentration of the standard solution was 15.00 mg L^−1^. The experimentally determined apocynin contents were calculated per 1 g of the dry sample and were statistically examined (Table 2). The value of the *RSD* (≤2.92%) indicates satisfactory precision of the developed procedure. The high recovery value for the spiked sample resulted from the total APO content. The amount of apocynin in the Kutki dietary supplement sample is approximately 1.36 mg/g. This was confirmed by the results obtained in the determinations in Kutki and Kutki spiked with known amounts of APO. Additionally, the apocynin content in Kutki was comparable to that determined using a CF microelectrode in an acetic acid environment [20]. Based on the data from the control assays, it can be concluded that the results obtained are reliable.

## 4. Conclusions

In this study, the mechanism of the anodic oxidation of apocynin on BDDE in phosphate buffer solutions (pH 7.3) has been described for the first time. This process is quasi-reversible, diffusion-controlled, and involves one electron and one proton. The APO radical, which is the primary product of the electrode reaction, is unstable and undergoes subsequent chemical transformations near the surface of the working electrode. The proposed mechanism of the process can thus be described as *E*_q_*C*_i_. The anodic oxidation of APO has become the basis for the development of a new voltammetric method to determine the analyte in natural samples. It was based on the DPV anodic peak at a potential of 0.605 V vs. Ag/AgCl. A good linear response was observed in the concentration range of 0.213–27.08 mg L^−1^. The estimated *LOD* and *LOQ* values were 0.071 and 0.213 mg L^−1^, respectively. These parameters are comparable to those obtained using chromatographic methods [16,17]. The effectiveness of the method was demonstrated using control determinations and analysis of APO in dietary supplement extracts. The developed procedure has been shown to be simple, rapid, sensitive, selective, and requiring no complicated sample preparation, which is limited only to a simple extraction with ethanol. The low consumption of non-toxic reagents makes it environmentally friendly and aligns with the principles of “green chemistry” [34]. A small amount of ethanol used as an extractant does not pose any threat to the environment. It also does not require special disposal of the resulting waste produced during the analysis. This procedure can also be the basis for APO determination in other matrices, for example, in plant, clinical, cosmetic, and pharmaceutical preparations. However, this requires additional studies to adapt the method to the properties and composition of the matrix. The method cannot be used in the presence of matrix components undergoing oxidation in a potential range similar to APO. It can also be easily used to determine other phenolic compounds with antioxidant properties in the above-mentioned matrices.

## Figures and Tables

**Table 1:** Comparison of analytical parameters for APO determination obtained with different techniques.

Method	Application	Linear Range (mg L^−1^)	*LOD* (mg L^−1^)	*LOQ* (mg L^−1^)	Ref.
HPLC-UV	rat plasma	0.2–100	nd ^1^	0.2	[16]
HPLC-PDA	bovine serum albumin nanoparticles	5–100	0.078	0.238	[17]
LC–MS/MS	rat and human plasma	1 × 10^−4^–1	nd ^1^	1 × 10^−4^	[18]
DPV (CF)	herbal extracts	0.449–43.2	0.148	0.449	[20]
DPV (BDDE)	herbal extracts	0.213–27.08	0.071	0.213	This work

^1^ No data.

**Table 2:** Results of the apocynin determination in control solution and dietary supplement by DPV.

Sample	APO Taken/mg L^−1^	^(1)^ APO Found/mg L^−1^	^(2)^*RSD*/% (n = 5)	^(3)^*R*/%
Control 1	0.2221	0.2212 ± 0.001	0.35	99.6
Control 2	0.9872	0.9959 ± 0.0095	0.77	100.9
Control 3	2.356	2.405 ± 0.034	1.13	102.1
Kutki	–	1.352 ± 0.042 mg g^−1^	1.85	–
Kutki with APO added	0.333 mg g^−1^	1.699 ± 0.06 mg g^−1^	2.92	510.2

^(1)^ x_av_ ± *t*_0.95_ *S*_av_ for n = 5 and *t*_0.95_ = 2.776 (tabulated), S_av_ denotes standard deviation of mean; ^(2)^ relative standard deviation; and ^(3)^ recovery, *R* = (x_av_/amount involved) × 100%.

## Data Availability

The original contributions presented in this study are included in the article. Further inquiries can be directed to the corresponding author.
